# Study of Emotional Effects of Tooth Loss in an Aging North Indian Community

**DOI:** 10.5402/2011/395498

**Published:** 2011-11-14

**Authors:** Amit Vinayak Naik, Ranjana C. Pai

**Affiliations:** ^1^Department of Prosthodontics, Teerthanker Mahaveer Dental College and Research Center, Uttar Pradesh, Moradabad, India; ^2^Teerthanker Mahaveer Dental College and Research Center, Uttar Pradesh, Moradabad, India

## Abstract

A study was conducted to study the emotional effects of complete and partial loss of teeth in an aging North Indian community. A questionnaire was prepared for 400 elderly people above the age of 60 years, who were interviewed in dental checkup camps. The data was collected and analyzed using chi-square or chi-square exact tests. 25% of the people were found to have difficulty accepting tooth loss, whereas more than 50% of the people reported to have restricted their food choices. Other problems like reduced attendance in social gatherings for lunch/dinner or eating out in public were also noted. 56% felt that dental consultation prior to tooth loss would have helped them in a significant way. It was concluded that tooth loss did not have a marked impact on emotions of the people but affected their daily social activities, however there was negligible difference between complete and partial tooth loss subjects.

## 1. Introduction

The loss of few or all natural teeth has been accepted as a handicapping condition for the aging elderly people. Apart from the negative emotions of normal aging, the loss of teeth adds to the emotional imbalance of the elderly [1–6]. Many studies have been conducted in the past to understand and cater the problems of tooth loss. These studies have concluded such subjects having less confidence, restricting social activities, and interpersonal relationships [7, 8]. Cultural differences and lifestyles have shown to affect the outcome of these studies. Countries of the east have shown to be more negatively influenced by tooth loss compared to their western counterparts [9, 10]. The awareness and availability of dental treatment and education were also a matter of concern in this issue. Hence a need was felt by the department of prosthodontics to conduct a study to understand the emotional effects of tooth loss in the elderly population of Northern India. Apart from this, a hypothesis of greater emotional effect for complete tooth loss (completely edentulous) compared to partial tooth loss (partially edentulous) was tested.

## 2. Method

The study involved 400 subjects aged between 60 to 80 years. The study excluded people suffering from systemic and psychological disorders and also people not oriented properly with respect to time, place, and person. 

A questionnaire was designed from the results of previous studies and investigations on emotional effects of tooth loss [7, 8]. A 24-open-ended questionnaire was designed in the colloquial Hindi language with space for any special comments for each question. The questions covered aspects of emotional disturbance, loss of confidence, social performance, productivity, and denture wear if any. The interview was conducted by only one trained dental officer in dental check camps in the locality of Uttar Pradesh (North India) to reduce interoperator variability. A self-assessment tooth-counting protocol including tooth counting and denture wear designed by Jepson et al. was used in the study. Denture wearers were asked regarding their denture use and the positive or negative effects of it in their mouths. 

Data was collected and analyzed. Comparisons were made between three groups, namely, completely edentulous, partially edentulous and weather denture wearers, and nondenture wearers for the first two groups. Variables including loss of confidence, acceptance of tooth loss, and restriction in activities were also analyzed. An independent chi-square or exact chi-square test was used to compare various distributions. The level of significance was set at 0.5. 

## 3. Results

A total of 400 elderly people aged between 60 to 80 years with complete or partial tooth loss were interviewed at 8 dental checkup camps in a locality in Northern India by a single dental officer. There was no statistically significant difference between the three dentition groups in the social, economic, and educational backgrounds of the participants (Table 1). 6% of the edentulous subjects had attended the dentist in the previous year compared with a third of the partially edentulous subjects (*P* < 0.001).

32% (128) of the subjects were edentulous and all of them wore complete denture. 35% (140) were partially edentulous (mean number of natural teeth was 11 with 7 SD and range from 1 to 28) and wore partial dentures, whereas 33% (132) were partially edentulous and were not wearing partial denture (mean number of natural teeth was 18 with 6 SD and range from 1 to 31) as shown in (Table 2).

### 3.1. Various Emotional Feelings Affected (Table 3)

**(1) Acceptance of Tooth Loss**

23% of the subjects had difficulty accepting tooth loss, 64% had no difficulty accepting tooth loss, and 13% were uncertain. There was no statistically significant difference between the three groups (*P* = 0.445). 35% accepted tooth loss immediately, 53% accepted it within 6 months, 5% within 1 year, 3% required more than 1 year, and 4% have still not accepted the loss. No difference was found in the time taken by the three groups in accepting the loss (*P* = 0.520).

**(2) Feelings about Tooth Loss**

60% of the subjects were indifferent and unconcerned about the tooth loss and 30% felt relieved. The partially edentulous subjects were seen to be more concerned and frightened than the other twogroups (*P* = 0.004). There was no difference between the groups for other emotional factors (*P* = 0.130  to  0.980).

**(3) Discussing Tooth Loss**

32% of subjects talked about tooth loss with others, among which 60% talked to their dentists, 55% talked to their friends, 31% talked to their spouses, and 55% talked with their family and relatives. There was no response between the groups (*P* = 0.1  to  0.8).

**(4) Wear of Dentures**

59% of the patients wore removable dentures, among them 18% avoided looking at themselves with dentures. There was no significant difference among the groups (*P* = 0.06  to  0.7). Partially edentulous subjects (80%) considered dentures as part of themselves compared with only 63% of edentulous subjects. (*P* = 0.012). 20% of edentulous group and 16% of denture wearing partially edentulous patients considered their denture to be a foreign body. 90% accepted the advice of not wearing the denture in the night, whereas 2% were indifferent and 8% found it unacceptable. No differences were found in the groups for the same (*P* = 0.05).

**(5) Preparation for Tooth Loss**

25% of the subjects were not prepared for the effects of tooth loss, 70% felt prepared, and the rest had no answer. More than half the people who were unprepared for the tooth loss felt that a consultation with the dentist along with education, motivation, and awareness would have reduced their problems.

**(6) Confidence**

96% did not feel any difference in their confidence, while the rest were indifferent. No difference was found between the groups (*P* = 0.19).

**(7) Restriction in Activities**

more than 50% of the subjects felt restricted with their food choices and enjoyment of food (*P* = 0.041), edentulous, and partially edentulous with dentures felt most restricted. Other feelings like eating in public, going out, laughing, and forming social relationships were of negligible importance. Going out was avoided by partially edentulous subjects who wore dentures (Table 4).

**(8)**

See Table 4

## 4. Discussion

The present study has been conducted according to the previous work done on the subject [9, 10]. Aging elderly people of Northern rural India formed the representative population for this study. Dental awareness and availability are generally low in this part. Elderly people turn up to the dentist only in pain or swelling. Hence a hypothesis was worked out stating that the emotional effects of tooth loss would be less compared to their counterpart privileged subjects. Majority of the subjects were indifferent or relieved by loss of teeth. The result shows that very few people were grieved by the loss of teeth as compared with other studies [9, 10]. 

The choice of food and enjoyment of food was negatively affected especially in complete edentulous and partial edentulous wearing dentures. This was directly proportional to the number of natural teeth remaining. The traditional Indian food in social gathering mostly consists of rotis, paranthas, and vegetables which require great masticatory efficiency. Hence loss of teeth imposes a handicapping situation for the aging elderly not only from the nutritional point of view affecting their physical health but also from the point of their overall mental wellbeing. Loss of teeth also means loss of esthetics in facial profile and personality, which does affect the social performance and ability of the individual to form social relations. But the psychosocial effects of tooth loss are less pronounced compared with other studies [9, 10]. This satisfaction after tooth loss also shows the less awareness, availability, and expectations of dental treatment to these elderly people. 

Quite a few subjects discussed tooth loss with family and friends showing the society's acceptance of tooth loss as normal aging procedure as compared to social stigma as compared to the western countries [9, 10]. Acceptance of the dentures was variable according to the availability and satisfaction of dental treatment. 

## 5. Conclusion

1. Loss of teeth did not have a marked emotional effect in the lives of the selected elderly community and had no difference between complete and partial edentulous subjects.
2. Significant disability and restrictions were seen in daily social activities.
3. Restrictions were more severe in people who had lost significant number of natural teeth requiring denture wearing.

## Figures and Tables

**Table 1:** Distribution of subjects (%) according to dentition with respect to age and gender.

		Edentulous (*n* = 128)	Partially edentulous with dentures (*n* = 140)	Partially edentulous without dentures (*n* = 132)	*P*
Age(years)	60–69	12	66	82	<0.001
	70+	88	34	18	<0.001
Gender	Male	41	65	55	0.047
	Female	59	35	45	0.047

**Table 2:** Distribution (%) of missing teeth in partially edentulous subjects.

Area	With removable dentures *n* = 140		Without removable dentures *n* = 132	
	Maxillary teeth	Mandibular teeth	Maxillary teeth	Mandibular teeth
				
All	26	18	2	2
Anterior	5	7	5	6
Posterior	22	28	66	68
Anterior + posterior	45	42	16	18
None	2	5	11	6

**Table 3:** Emotional feelings associated with tooth loss (%).

Emotions	Edentulous (*n* = 128)	Partially edentulous with dentures (*n* = 140)	Partially edentulous without dentures (*n* = 132)	*P*
Unconcerned/indifferent	62	35	49	0.004
Scared/frightened	14	33	14	0.004
Handicapped feeling	3	3	5	0.6
Feeling old	1	4	4	0.3
Relieved	28	31	29	0.9
Angry	1	2	3	0.8
Resigned	4	9	9	0.4
Sad/depressed	0	1	0	0.5
dismayed	10	20	22	0.13
This is not happening to me	0	0	1	0.5

**Table 4:** Restriction in activities (%).

Restriction	Edentulous (*n* = 128)	Partially edentulous with dentures (*n* = 140)	Partially edentulous without dentures (*n* = 132)	*P*
Restricted food choice	61	63	45	0.04
Avoid eating out	6	13	12	0.16
Avoid going out	5	11	2	0.2
Enjoyed food less	52	60	30	<0.001
Avoided laughing	6	13	12	0.29
Avoided social Relationships	3	8	6	0.39
